# Proteases HtrA and HtrB for α-amylase secreted from *Bacillus subtilis* in secretion stress

**DOI:** 10.1007/s12192-019-00985-1

**Published:** 2019-04-18

**Authors:** Shaomin Yan, Guang Wu

**Affiliations:** State Key Laboratory of Non-Food Biomass and Enzyme Technology, National Engineering Research Center for Non-Food Biorefinery, Guangxi Key Laboratory of Bio-refinery, Guangxi Academy of Sciences, 98 Daling Road, Nanning, 530007 Guangxi China

**Keywords:** α-Amylase, *B. subtilis*, HtrA, Secretion stress, Sigma factor

## Abstract

HtrA and HtrB are two important proteases across species. In biotechnological industries, they are related to degradation of secreted heterologous proteins from bacteria, especially in the case of overproduction of α-amylases in *Bacillus subtilis*. Induction of HtrA and HtrB synthesis follows the overproduction of α-amylases in *B. subtilis*. This is different from the order usually observed in *B. subtilis*, i.e., the production of proteases is prior to the secretion of proteins. This discrepancy suggests three possibilities: (i) HtrA and HtrB are constantly synthesized from the end of the exponential phase, and then are synthesized more abundantly due to secretion stress; (ii) There is a hysteresis mechanism that holds HtrA and HtrB back from their large amount of secretion before the overproduction of α-amylases; (iii) Heterologous amylases could be a stress to *B. subtilis* leading to a general response to stress. In this review, we analyze the literature to explore these three possibilities. The first possibility is attributed to the regulatory pathway of CssR-CssS. The second possibility is because sigma factor σ^D^ plays a role in the overproduction of α-amylases and is subpopulation dependent with the switch between “ON” and “OFF” states that is fundamental for a bistable system and a hysteresis mechanism. Thus, sigma factor σ^D^ helps to hold HtrA and HtrB back from massive secretion before the overproduction of α-amylases. The third possibility is that several sigma factors promote the secretion of proteases at the end of the exponential phase of growth under the condition that heterologous amylases are considered as a stress.

## Introduction

Secretion stress usually refers to the high-level α-amylase production in *Bacillus subtilis* (Lulko et al., 2007; Ploss et al., 2016) although it was also observed in the overproduction of Sec-secreted proteins in *Streptomyces lividans* (Gullón et al., 2012; Vicente et al., 2016). Secretion stress is largely dependent upon the nature of the secreted protein that is overproduced (Westers et al., 2006). Secretion stress goes through three different systems: Sec secretion system, CssR-CssS regulatory system, and HtrA and HtrB proteases. Sec system is a component in type II secretion system and is involved in secretion stress (Yan and Wu, 2017). CssR-CssS stands for controlling regulator and sensor of secretion stress, and is a two-component system (Hyyryläinen et al., 2001) belonging to class V heat-inducible genes (Darmon et al., 2002). HtrA and HtrB are two membrane-bound proteases that were initially termed as membrane-bound HtrA-like proteases YkdA and YvtA (Noone et al., 2000; Tjalsma et al., 2000).

**Fig. 1:**
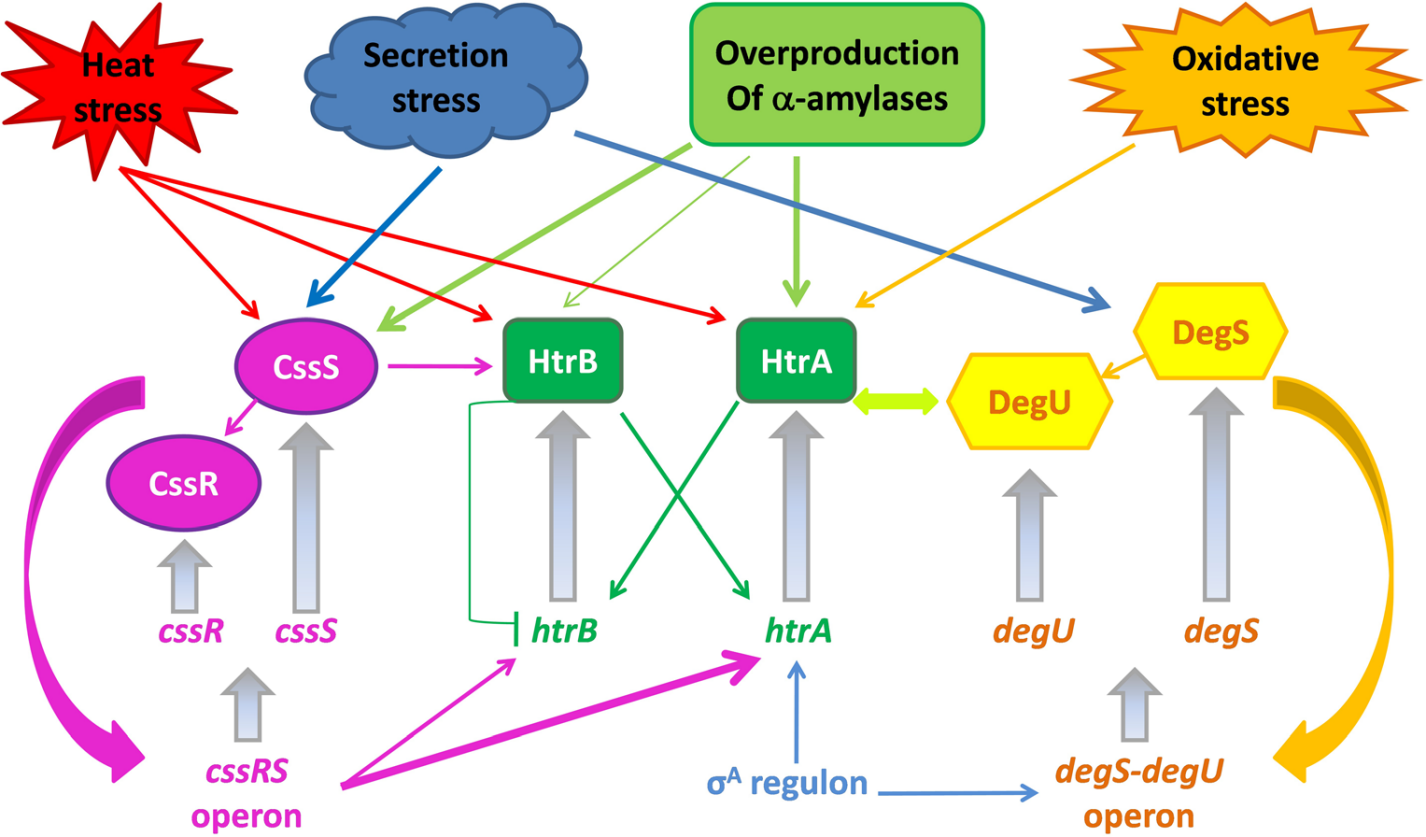
Secretion stress due to overproduction of α-amylases in *B. subtilis* and induction of HtrA and HtrB synthesis with various stresses and regulators. An arrow indicates a positive regulation, an ending symbol indicates a negative regulation, and the line width is proportional to regulatory effect

In this review, we are particularly interested in HtrA and HtrB (Fig. 1), because they degrade α-amylases (Darmon et al., 2002; Ploss et al., 2016) that are heterogeneously expressed and secreted from *B. subtilis*. At first glance, the pathway of secretion stress looks simple; however, its regulation includes not only the auto-regulation of *cssRS* operon but also the cross-regulation (pink colored items in Fig. 1). This auto-regulation is subject to the level of CssR and CssS, absence of HtrA or HtrB, heat, and secretory proteins (Darmon et al., 2002; Noone et al., 2000, 2001). HtrA and HtrB have the reciprocal cross-regulation of their own genes and a negative auto-regulation of *htrB* (green-colored items in Fig. 1) (Noone et al., 2001). The regulation of HtrA and HtrB, especially for HtrA, is complicated, and has been the subject of reviews with their three-dimensional structure (Clausen et al., 2011; Hansen and Hilgenfeld, 2013; Pallen and Wren, 1997; Singh et al., 2011).

For HtrA, our knowledge largely comes from the studies in Gram-negative bacteria, whose proteases are better characterized than their counterparts in Gram-positive bacteria. For example, DegP from *Escherichia coli* is a model protease of the HtrA family. Two trimeric HtrA rings form a hexamer in the inactive state (Krojer et al., 2002), while the hexamers are dissociated into trimers in the active state (Krojer et al., 2008). By contrast, our knowledge on HtrA homologs in Gram-positive bacteria is limited.

**Fig. 2:**
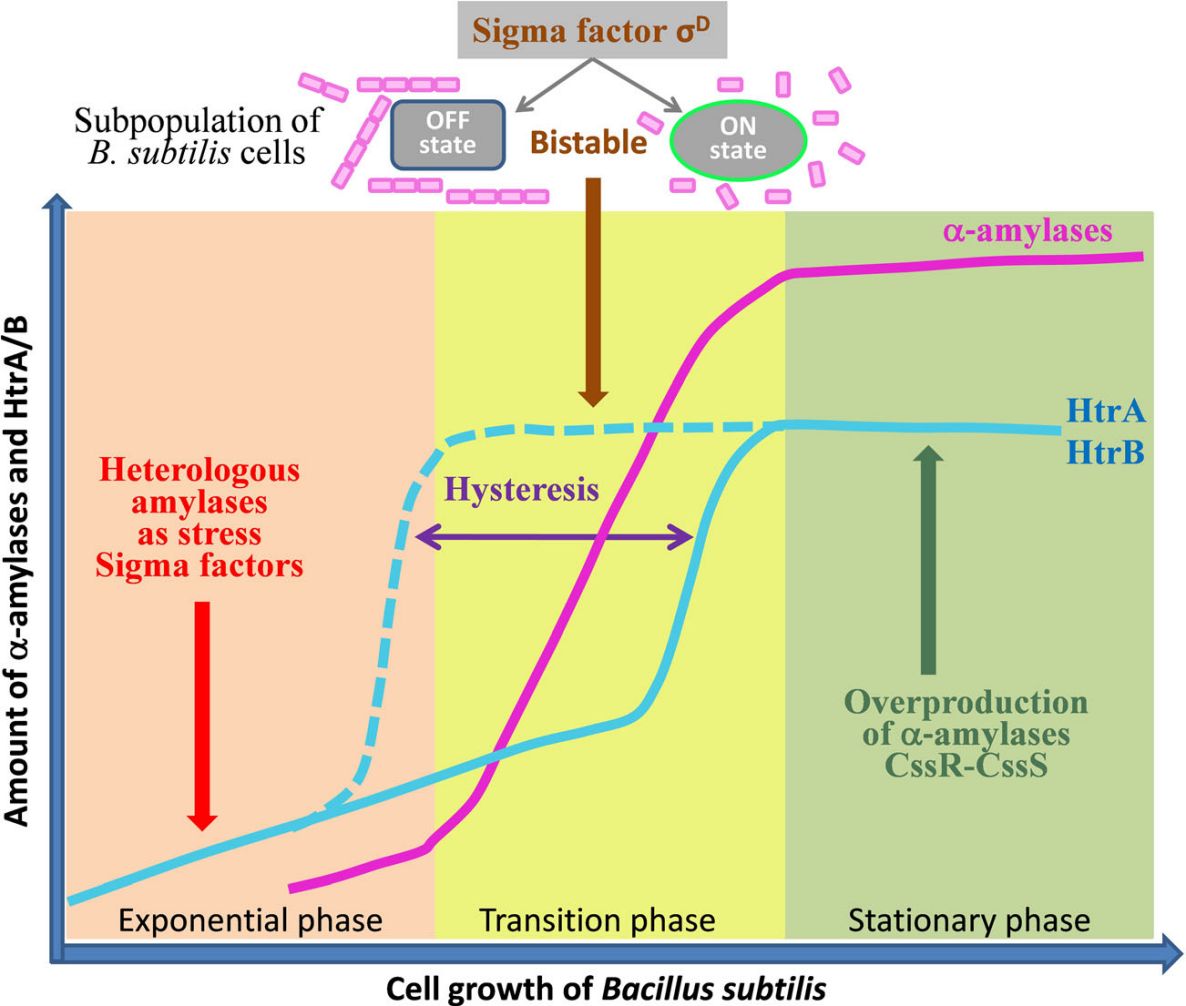
Production of α-amylases and induction of HtrA and HtrB synthesis along the time course with respect to possible hysteresis

The well-known secretion stress is very specific because the induction of HtrA and HtrB synthesis comes from the overproduction of α-amylases in *B. subtilis* (light green-colored rectangle on the top of Fig. 1)*.* Hence, the overproduction of α-amylases occurs prior to the synthesis of HtrA and HtrB. This is interesting, because the synthesis of proteases, including HtrA and HtrB (Gullón et al., 2012), often occurs at the end of the exponential phase of growth in *Bacillus* (Margot and Karamata, 1996; Priest, 1977; Shen et al., 2005), especially when a large amount of proteases is needed (Liu et al., 2005) (solid blue line in Fig. 2). On the other hand, most secretion of proteins in *B. subtilis* occurs at the beginning of the stationary phase of growth (Bolhuis et al., 1998; Herbort et al., 1999). Generally, the activity of secretion of proteins is quite low during exponential phase but increases substantially at the onset of stationary phase (Priest, 1977). Moreover, several components of Sec secretion system, through which α-amylases are secreted, reach their maximum expression either at the end of exponential growth phase (Herbort et al., 1999) or at the early post-exponential phase (Bolhuis et al., 1998) in *B. subtilis* (pink line in Fig. 2).

This discrepancy highlights a hysteresis feature (dashed blue line in Fig. 2) suggesting three possibilities: (i) HtrA and HtrB are constantly synthesized from the end of the exponential phase, and are then synthesized more abundantly due to secretion stress; (ii) There is a hysteresis mechanism that holds HtrA and HtrB back from a large amount of secretion before the overproduction of α-amylases; (iii) Heterologous amylases could be a stress to *B. subtilis* leading to a general response to stress because they can induce HtrA and HtrB synthesis at the transition phase of the growth cycle (Noone et al., 2001) or at the late exponential phase and the stationary-growth phase (Lulko et al., 2007). Hence, the aim of this review is designed to explore these possibilities by reviewing literature.

## Induction of synthesis of HtrA and HtrB by CssR-CssS due to overproduction

HtrA and HtrB stand for high-temperature requirement A and B (Darmon et al., 2002), and their expression is induced by heat stress and α-amylase overproduction (Noone et al., 2000, 2001), but CssR-CssS controls their induction (lower left side in Fig. 1). However, the response of both HtrA and HtrB to CssR-CssS reveals a difference, in that HtrA is more sensitive to CssR-CssS (Hyyryläinen et al., 2001). As HtrA and HtrB are inducible by α-amylase overproduction, our question raised here is which one induces the synthesis of HtrA and HtrB: the misfolded α-amylases or the quantity of overproduced α-amylases? It seems that misfolded α-amylases might not be entirely related to the overproduction of α-amylases.

### CssS as a sensor

As a bicomponent regulatory system, CssR and CssS work as the response regulator CssR and the membrane-embedded sensor kinase CssS. The CssS detects misfolded proteins at the membrane–cell wall interface and monitors the overproduction of α-amylase (AmyQ), and in return controls the synthesis of extracytoplasmic proteases. The activation of CssR goes through phosphorylation (Hyyryläinen et al., 2001). However, the signal to induce *htrA* and *htrB* expression is probably not misfolded cytosolic proteins, because puromycin is unable to induce their expression (Darmon et al., 2002; Noone et al., 2000). Still, the induction of *htrB* is CssS dependent for secretion stress whereas the induction of *htrA* and *htrB* is CssS dependent for heat stress (left side in Fig. 1). Actually, *cssRS* responds to high levels of α-amylases (Darmon et al., 2002).

HtrA is not the only protease whose synthesis is controlled by the CssR-CssS system at the membrane–cell wall interface (Hyyryläinen et al., 2001), because the results from *cssS* and *htrA* mutant strains suggested that CssR-CssS also controls at least another protease (Darmon et al., 2002). On the other hand, CssR-CssS does not regulate all proteases. For example, a membrane–cell wall interface serine protease CWBP52 in *B. subtilis* is beyond its regulation (Margot and Karamata, 1996). In fact, neither the nature of heterologous amylases nor their secretion was sufficient for *htrA* and *htrB* induction. Their induction includes (i) the secretion load such as the total number of proteins and the amount of each protein being processed and/or secreted, (ii) the stage of protein maturation, and (iii) the degradation level of aberrant protein (Noone et al., 2001). As the misfolded α-amylases may not be directly linked with the overproduction of α-amylases, CssS serves as a sensor to detect the overproduction of α-amylases.

As a protease induced by heat shock (red-colored items in Fig. 1), HtrA belongs to serine proteases and exists widely across species (Pallen and Wren, 1997). The study on extracellular proteome of *B. subtilis* demonstrated two types of HtrA (Antelmann et al., 2003): membrane-bound HtrA and soluble HtrA. The former one has an N-terminal membrane anchor whereas the transmembrane region is cleaved in the latter one. The secretion mechanism for HtrA in *B. subtilis* is similar to the human HtrA2/Omi protein (Vande Walle et al., 2008), i.e., the full-length HtrA is firstly transported and integrated into cytoplasmic membrane, then is processed into a soluble form with activity, and finally is released into the extracytoplasmic space.

### HtrA as chaperone

Two locations of HtrA in *B. subtilis* imply its two functions (Antelmann et al., 2003). In addition to the protease activity, HtrA acts as a molecular chaperone when present in the culture supernatant (Antelmann et al., 2003). Also, HtrA works as a chaperone at lower temperatures but as a protease at high temperature (Spiess et al., 1999). In such a case, HtrA would have a similar function as DnaK and GroEL to prevent the accumulation of unfolded proteins during stress (Wegele et al., 2004). The overproduction of α-amylases leads to a stimulatory effect on the genes for the general stress proteins, DnaK and GroEL (Lulko et al., 2007). Therefore, HtrA, that is synthesized at the end of the exponential phase, could function as a chaperone to deal with misfolded α-amylases.

Furthermore, the study on extracellular proteome of *B. subtilis* showed that only HtrA and YqxI had changed in response to secretion stress (Antelmann et al., 2003). Under the condition of high-level AmyQ production, an augmentation of transcription can be found in *htrA* but not in *yqxI*. The amount of extracellular HtrA increases at the transcriptional level, whereas that of YqxI changes at post-transcriptional level and is HtrA dependent. However, no significant change was found in extracellular amount of HtrB in *B. subtilis* in response to secretion stress (Antelmann et al., 2003). Therefore, there are substantial amounts of HtrA near the extracytoplasmic membrane surface of *B. subtilis* under the condition of secretion stress. This suggests that HtrA in *B. subtilis* may serve as a detector sensing secretion stress like CssS (Hyyryläinen et al., 2001).

Crystallographic study illustrates the structure of the PDZ domain of HtrA that contains trimeric rings forming flexible sidewalls with a central cavity. The PDZ domain is considered necessary for proteolytic function and/or chaperone-like function because it binds to its substrate and brings it into the cavity (Krojer et al., 2002). Intriguingly, the HtrA from *E. coli* has two PDZ domains whereas the HtrA from *B. subtilis* has only one PDZ domain. This implicates that a single conserved PDZ domain in *B. subtilis* HtrA is sufficient for its dual functions (Antelmann et al., 2003). However, multiple PDZ domains in the HtrA from *Thermotoga maritima* appear dispensable to perform both proteolytic and chaperone-like functions (Kim and Kim, 2002).

### HtrA activation

The main mechanism for activating the HtrA proteases is based on allostery as well as temperature (Figaj et al., 2014). Binding of an appropriate allosteric peptide or a substrate leads to structural rearrangement of the protein and consequently to increased activity of the enzyme (Hansen and Hilgenfeld, 2013; Löwer et al., 2008). However, additional mechanisms exist. For example, the HtrA (DegP) in *E. coli* may be regulated by reversible reduction/oxidation of the S-S bridge (Figaj et al., 2014; Koper et al., 2015) located in the regulatory loop LA (Skorko-Glonek et al., 2006).

The review in this section supports the possibility that HtrA and HtrB may be constantly synthesized from the end of the exponential phase, and then synthesized more abundantly due to secretion stress through CssR-CssS regulatory pathway.

## Induction of synthesis of HtrA and HtrB by sigma factor σ^D^

As HtrA is independent of CssR in *B. subtilis* (Lulko et al., 2007), our question raised here is whether *B. subtilis* has a secondary mechanism to induce the synthesis of HtrA and HtrB.

Sigma factor σ^D^ is expressed during vegetative growth and controls the expression of genes during exponential growth and the early stationary phase (Marquez et al., 1990). Sigma factor σ^D^ is related to the gene expression of flagellum and motility, whose upregulation is an adaptive response in *B. subtilis* (Helmann et al., 1988; Marquez et al., 1990). Upon AmyQ overproduction, motility-specific (σ^D^-dependent) transcripts were upregulated (Lulko et al., 2007). Some flagellar genes can only express in a subpopulation of *B. subtilis* (Kearns and Losick, 2005), whose population bifurcates into two types (top part in Fig. 2). During exponential growth phase, *B. subtilis* cells can appear either single motile individuals or sessile cell chains, where the cells join end-to-end (Mukherjee and Kearns, 2014). This population heterogeneity is under the control of sigma factor σ^D^ (Chen et al., 2008; Helmann et al., 1988; Kearns and Losick, 2005; Marquez et al., 1990).

### Evidence from σ^D^

In biotechnological settings, a flagellum gene, *hag*, is knocked out from *B. licheniformis* BL9 and BL10 in order to increase the yield of α-amylase (Chen et al., 2015) and nattokinase (Wei et al., 2015). Also *hag* can serve as a reporter to study environmental effects on σ^D^-dependent gene expression (Lulko et al., 2007; Mirel and Chamberlin, 1989). At the “ON” state for sigma factor σ^D^ activity, *B. subtilis* can express *hag* that completes flagellum assembly, and *lytF* that is a peptidoglycan endopeptidase (Yamamoto et al., 2008; Margot et al., 1999) and separates the cells from chains. At the “OFF” state for sigma factor σ^D^ activity, neither *hag* nor *lytF* is expressed in *B. subtilis* so the cells form non-motile chains (Chen et al., 2008). Therefore, the activity of sigma factor σ^D^ decides the fate of individual cells in each subpopulation (Mukherjee and Kearns, 2014). The strains commonly used in laboratories are biased towards the OFF state and grow predominantly as long chains, whereas the ancestral strain is biased towards the ON state and grow predominantly as motile individuals (Kearns and Losick, 2005). As a result, the *Bacillus* strain, whose *hag* is knocked out, is highly likely to belong to the OFF state. Our question raised here is what is the population heterogeneity for *B. subtilis*-producing α-amylase? Consequently, does the portion of α-amylase-producing *B. subtilis* cells belong to the ON or OFF state?

### Hysteresis

Evidence suggests that there is a hysteresis mechanism that holds HtrA and HtrB back from a large amount of secretion prior to the overproduction of α-amylases. Essentially, the hysteresis is a characteristic of bistable systems (top part in Fig. 2), where an acquired state resists to switch into another state in the absence of a history-dependent stimulus (Priest, 1977). For this reason, we can consider the ON and OFF states for sigma factor σ^D^ activity as a bistable system.

### Further evidence from σ^D^

In *B. subtilis*, DegU-P can activate promoter *flgM* (Hsueh et al., 2011), whose product FlgM inhibits sigma factor σ^D^ (Bertero et al., 1999; Caramori et al., 1996; Daughdrill et al., 1997; Sorenson et al., 2004). SwrA (swarming motility protein) may activate the *fla*/*che* operon indirectly by binding to and antagonizing a repressor, the phosphorylated form of the response regulator DegU-P (Amati et al., 2004; Mordini et al., 2013; Ogura and Tsukahara, 2012; Tsukahara and Ogura, 2008), although this protein is discontinued in gene bank for *B. amyloliquefaciens* FZB42, LL3, TA208, XH7, Y2, etc. For sigma factor σ^D^, its feed-forward regulation is the reason for bistability (Allmansberger, 1997; Estacio et al., 1998; Mordini et al., 2013) that could lead to the abovementioned hysteresis for HtrA and HtrB. Moreover, sigma factor σ^D^ increases during growth and reaches its maximum level at the transition point (Mirel and Chamberlin, 1989; Mirel et al., 2000), whose trend is similar to the induction of HtrA and HtrB at the transition phase of the growth cycle (Noone et al., 2001) or in the late exponential phase and the stationary-growth phase (Lulko et al., 2007). And the overproduction of α-amylases prolongs the motile phase in *B. subtilis* (Lulko et al., 2007), i.e., the ON state, which could lead to replacement and competition for core RNA polymerase during stationary phase in *B. subtilis* (Hicks and Grossman, 1996; Ju et al., 1999; Lord et al., 1999). Furthermore, these can be connected to sporulation (Eichenberger et al., 2004), and RNA polymerases in excess could lead to deactivation of σ^A^ (middle lower part in Fig. 1) (Fujita, 2000).

To this end, the induction of synthesis of HtrA and HtrB is the consequence of activation of CssR-CssS by peptidoglycan recognition proteins. The layer of peptidoglycan is thicker in *B. subtilis* than in other bacteria (Mukherjee and Kearns, 2014). Peptidoglycan recognition proteins can depolarize the cell membrane, stop synthesizing intracellular peptidoglycan, protein, RNA, and DNA, but they can also produce hydroxyl radicals leading to bacterial death in *B. subtilis* (Kashyap et al., 2011). In this regard, the activity of sigma factor σ^D^ on *lytF*, peptidoglycan endopeptidase (Margot et al., 1999; Yamamoto et al., 2008), could explain the link between HtrA and oxidation.

The review in this section explores the possibility that the synthesis of HtrA and HtrB is induced by the overproduction of α-amylases through sigma factor σ^D^, and the possibility that sigma factor σ^D^ helps to hold HtrA and HtrB back from massive secretion before the overproduction of α-amylases that is the mechanism of hysteresis.

## Possible roles of other sigma factors

Since the induction of the synthesis of HtrA and HtrB by secretion stress in *B. subtilis* occurs quite late, our question raised here is whether *B. subtilis* has other mechanisms to induce HtrA and HtrB synthesis before the secretion stress? An answer discussed above is that the induction of HtrA and HtrB synthesis before the secretion stress is to use HtrA as a chaperone. However, some studies suggested that HtrA is more likely to function as a protease rather than chaperone in *B. subtilis* (Margot and Karamata, 1996) and in *E. coli* (Chang, 2016; Ge et al., 2014).

Thus, we need to explore the third possibility, i.e., do heterologous amylases serve as a stress to *B. subtilis*? If this is so, then heterologously expressed proteins could induce the general response to stress. Truly, *B. subtilis* has a sophisticated regulatory system in response to various stresses and maintains its survival, for which the induction of general response proteins is the earliest response (Hecker and Völker, 2001). Well-known stresses like heat shock (Hyyryläinen et al., 2001) (left upper corner in Fig. 1) and oxidation (Kashyap et al., 2011) (right upper corner in Fig. 1) can induce HtrA in *B. subtilis* because HtrA belongs to the regulon that is linked to the response to oxidative stress in *B. subtilis* (Noone et al., 2000). Once again, our attention is given to sigma factors, not only because sigma factors in *B. subtilis* play crucial roles at different levels in response to stresses but also because heterologous amylases are a stress.

### Sigma factor σ^A^

At least three well-characterized classes of heat-inducible genes exist in *B. subtilis*, i.e., HrcA/CIRCE (Hecker et al., 1996; Zuber and Schumann, 1994), SigB (Hecker and Völker, 1998) and CtsR (Derré et al., 1999; Krüger and Hecker, 1998), and sigma factor σ^A^ (middle lower part in Fig. 1). The σ^A^ is found from purified RNA polymerase (Shorenstein and Losick, 1973) and belongs to group I sigma factor (Haldenwang, 1995; Gruber and Gross, 2003). It deals with the expression of genes responding to heat shock (Chang et al., 1994), especially when *B. subtilis* cells grow in rich medium (Haldenwang, 1995). In fact, the overexpression of heterologous proteins was considered as a stress to *B. subtilis* (Mogk et al., 1998) because folded or misfolded proteins can trigger the cellular stress response when their number increases. HrcA represses the genes encoding chaperones, but its function is influenced by chaperones, for example, HrcA activation needs GroE (Mogk et al., 1997). During heat stress or protein overproduction, the chaperones deal with accumulated proteins, resulting in HrcA inactivation. Additionally, oxidative stress indirectly induces misfolded proteins, leading to the expression of chaperone encoding genes that are regulated by CtsR (Derré et al., 1999). DegS belongs to a two-component regulatory system DegS-DegU regulating the synthesis of many secretory enzymes and being subject to sigma factor σ^A^ (Msadek et al., 1990) (right lower part in Fig. 1). In fact, the mutations in *degS* and *degU* lead to the overproduction of proteases (Msadek et al., 1990; Tanaka et al., 1991). Interestingly, special attention has been given to the role of DegU in the overproduction of α-amylases in *B. subtilis* (Ploss et al., 2016). Also, the level of DegU-DegP was correlated with noisy transcription of subtilisin, *aprE* in *B. subtilis* (Veening et al., 2008).

### Sigma factor σ^B^

In *B. subtilis*, more than 40 genes belong to class II general stress genes, and sigma factor σ^B^-dependent promoters induce their expression in various conditions of stress (Hecker et al., 1996). Sigma factor σ^B^ is the first alternative sigma factor discovered in bacteria, and controls more than 150 genes (Hecker and Völker, 2001). These general stress proteins can protect non-growing cells by transforming them to be resistant to the damages from non-specific and multiple stresses, including acid, alkaline, heat, osmotic stress, or oxidative stress. Such an essential response to mild stresses may prevent cell death after future potentially lethal stress. Thus, the response of sigma factor σ^B^ is considered as a strategy of alternative survival, where the non-growing cell can stay in a vegetative state rather than sporulative state (Hecker and Völker, 2001). The main function of the σ^B^ regulon is protecting *B. subtilis* from reactive oxygen radicals. The activation of σ^B^-dependent transcription occurs during the stationary growth of *B. subtilis* cells or when they encounter some unfavorable environmental conditions, such as ethanol, heat shock, high salt, or O_2_ limitation (Haldenwang, 1995). The activity of sigma factor σ^B^ is controlled by RsbV-RsbW pathway, where RsbV is an anti-anti-sigma factor and binds to RsbW so sigma factor σ^B^ is activated (Alper et al., 1996). RsbW is an anti-sigma factor and binds to sigma factor σ^B^ to prevent the formation of holoenzyme E-σ^B^ (Bensom and Haldenwang, 1993), and RsbW is hypothesized to be sensitive to the ratio of ATP/ADP because ATP decrease can induce σ^B^ (Haldenwang, 1995).

### Sporulation-specific factors

The sporulation in *B. subtilis* is characterized by cascades of gene expression that are regulated by sigma factors σ^E^, σ^F^, σ^G^, and σ^K^ (Stragier and Losick, 1990). Sigma factor σ^E^ plays an important role in *B. subtilis* sporulation (Feucht et al., 2003; Chary et al., 2005), and regulates the expression of many sporulation genes, such as SpoIIT (Meeske et al., 2016). The overproduction of α-amylases can inhibit the sporulation in *B. subtilis* (Lulko et al., 2007) either by downregulating *spo0A* or by upregulating *spo0JA* and *spo0E* that negatively affect sporulation (Piggot and Hilbert, 2004). Indeed, sporulation can be initiated not only by starvation but also by various factors, including flagellar motility, production of antibiotics, secretion of proteases, heat stress, etc. (Meeske et al., 2016). An interesting question here is whether the induction of HtrA and HtrB synthesis provides the signal for the formation of heat-resistant spores? In order to prevent the formation of heat-resistant spores, the inhibition of sporulation happens (Lulko et al., 2007). SpoIIT functions directly in the cell–cell signaling pathway that triggers proteolytic activation of sigma factor σ^E^ (Meeske et al., 2016). Initially, an inactive membrane-associated precursor pro-σ^E^ is synthesized (LaBell et al., 1987; Zhang et al., 1998), and then pro-σ^E^ undergoes a proteolytic process to become sigma factor σ^E^ (Fujita and Losick, 2002; Hofmeister, 1998).

In dormant spores of *B. subtilis*, there are 10 to 20% of the proteins belonging to small, acid-soluble spore proteins, and they are encoded by genes *sspA* to *sspE*. One of the genes transcribed by E-σ^F^ is *gpr* that encodes an endopeptidase to degrade small, acid-soluble spore proteins as a source of amino acids when spore germination occurs (Sussman and Setlow, 1991). SpoIIAA/SpoIIAB regulatory system determines when and where to activate sigma factor σ^F^. SpoIIAB preferentially binds to sigma factor σ^F^ when a cell has high ATP/ADP ratio, whereas SpoIIAB preferentially binds to SpoIIAA when a cell has low ATP/ADP ratio (Haldenwang, 1995). In the latter condition, SpoIIAB phosphorylates SpoIIAA, and thus inhibits the binding of SpoIIAA to SpoIIAB.

The holoenzyme E-σ^G^ takes part in the transcription of *sspE* and *gpr* (Sussman and Setlow, 1991). In mutant *B. subtilis*, *gpr* transcription depends first on E-σ^F^, and then on E-σ^G^. In reality, *gerA* and *gerD* are a part of the σ^G^ regulon, indicating the involvement of sigma factor σ^G^ in germination of *B. subtilis*.

The synthesis of sigma factor σ^K^ in *B. subtilis* reveals more complicated regulation than that of other sigma factors (Haldenwang, 1995). Overexpression of cytoplasmic proteins could either have no effect on early sporulation gene expression (Jurgen et al., 2001) or have an effect on early sporulation gene expression (Lulko et al., 2007). The former suggests that the stress due to heterologous proteins rather than the stress due to overproduction would downregulate sporulation genes, whereas the latter suggests that both stresses play their role in early sporulation gene expression (Lulko et al., 2007). So our question raised here is which leads to the inhibition of sporulation, CssS or HtrA?

### The extracytoplasmic function family

The extracytoplasmic function family belongs to group IV sigma factors, including σ^M^, σ^V^, σ^W^, σ^X^, σ^Y^, σ^Z^, and YlacZ (Helmann, 2002) in *B. subtilis*. They function with corresponding transmembrane anti-sigma to control some genes that relate to cell surface or transport (Gruber and Gross, 2003), so they regulate the integral response to cell envelope stresses and play roles to maintain homeostasis of the cell envelope (Helmann, 2016). Until now, there is lack of information on direct evidence between the group IV sigma factors and the overproduction of α-amylases.

The review in this section explores the possibility that heterologous amylases are a stress to *B. subtilis*, and therefore, the synthesis of HtrA and HtrB before the secretion stress could be attributed to the general response operated by sigma factors in *B. subtilis*. In particular, sigma factors σ^A^ and σ^B^ could be responsible to the induction of HtrA and HtrB synthesis before the secretion stress, whereas sigma factors σ^E^, σ^F^, σ^G^, and σ^K^ could participate in the induction of HtrA and HtrB synthesis due to the overproduction of α-amylases through a sporulation mechanism.

## Conclusions

In this review, we analyze the literature to address the relationship between the secretion stress due to α-amylases overproduction and the induction of HtrA and HtrB synthesis. The results shed light on understanding the discrepancy, i.e., in the common concept that the synthesis of proteases often occurs at the end of the exponential phase of growth in *Bacillus*, while most of protein secretion in *B. subtilis* occurs at the beginning of the stationary phase of growth.

The literature review supports the notion that the increase of HtrA and HtrB comes from the overproduction of α-amylases through CssR-CssS regulatory pathway (Fig. 1). The literature review suggests that sigma factor σ^D^ also plays a role in the overproduction of α-amylases that may be subpopulation dependent, and sigma factor σ^D^ may help to hold HtrA and HtrB back from massive secretion before the overproduction of α-amylases (Fig. 2). The literature review implies that several sigma factors can promote the secretion of proteases at the end of the exponential phase of growth under the condition that heterologous amylases are considered as a stress.

Finally, it is curious why a cell must eliminate its secreted misfolded proteins in its extracellular space? In general, these secreted misfolded proteins are cut into pieces by secreted proteases, and then the fragments are taken into the cell. These fragments were supposed to facilitate efficient initiation of degradation by the proteases (Inobe and Matouschek, 2014; van der Lee et al., 2014). Does this suggest the lack of nutrients during the exponential phase for the cell? Furthermore, proteins with high degree of intrinsic disorder have a short cellular half-life (Tompa et al., 2008). How long is the half-life of α-amylase in extracellular space? Nevertheless, the answers to these questions would be helpful for better operation of microbial cell factories.
